# Japanese insurers’ attitudes toward adverse selection and genetic discrimination: a questionnaire survey and interviews with employees about using genetic test results

**DOI:** 10.1038/s10038-020-00873-y

**Published:** 2020-11-11

**Authors:** Hiroshi Iida, Kaori Muto

**Affiliations:** 1 Department of Computational Biology and Medical Sciences, Graduate School of Frontier Sciences, The University of Tokyo, Tokyo, Japan; 2 Department of Public Policy, The Institute of Medical Science, The University of Tokyo, Tokyo, Japan

**Keywords:** Ethics, Economics

## Abstract

Since the 1990s, insurance has been the primary field focused on the social disadvantages of using genetic test results because of the concerns related to adverse selection. Although life insurance is popular in Japan, Japan does not currently have any regulations on the use of genetic information and insurers have largely kept silent for decades. To reveal insurers’ attitudes on the topic, we conducted an anonymous questionnaire survey with 100 insurance company employees and recruited nine interviewees from the survey respondents. We found that genetic discrimination is not generally considered as a topic of human rights. We also found that insurers have uncertain fears and concerns about adverse selection in terms of actuarial fairness but not regarding profits. When it comes to preparing guidelines on the use of genetic information by Japanese insurers, we believe that public dialog and consultation are necessary to gain understanding of the people.

Since the 1990s, insurance has been the primary field focused on the social disadvantages of using genetic test results [1]. There is a possibility that people whose genetic test results show that they are predisposed to certain future illnesses may be at a disadvantage in acquiring the necessary insurance or that they may be denied insurance altogether. As opposed to this, from the insurers’ point of view, if the number of applicants who hide their future illnesses increase, it could damage the actuarial benefits due to adverse selection [2–4] and also lead to additional insurance claims as a result of intentional murder or suicide. Adverse selection means the applicants’ attitudes toward enrolling for intentional claims or hiding their disadvantageous information such as current illness to acquire insurance; this is against their obligation to disclose material information under situations wherein insurers lack access to applicant data due to asymmetric information [5]. However, in many countries, insurers’ use of genetic information for risk selection is regarded as genetic discrimination [6–9], and this practice continues. In the UK, the moratorium agreement between the government and the insurance association banning insurers from using genetic test results, except for a large amount of insurance, was renewed in October 2018 [10]. In Australia, insurers had been allowed to use genetic test results on an actuarial basis. However, there was a debate that it was illegal and discriminatory that the insurers did not change their underwiring terms even after surgery to reduce the risk of onset [11]. In July 2019, the Australian government steered to prohibit insurers from using genetic test results [12].

By contrast, while Japan does not currently have any regulations on the use of genetic information for risk assessment purposes, Japanese insurers have explained that they have refrained from using such information for insurance. Under the universal health coverage provided to all citizens since 1961, private Japanese life insurance firms primarily sell death benefit packages to protect the bereaved; these products include whole life insurance, which is sold door to door. Japan has the world’s highest average life insurance value, at about USD 210,000 per household, and 88% of households hold life insurance [13]. Death benefits account for ~65% of all Japanese life insurance products [13]. Even though private life insurance in Japan is literally referred to as a “safety net” in recent decades, Japanese insurers have largely kept silent on the use of genetic test results, and the government has not provided any opportunities for policy debates on the matter.

Although some studies have examined insurers’ views on the use of genetic information [14, 15], only one has done so in a Japanese context [16]. It revealed that current declarations do not require genetic information. Our study aims to reveal insurers’ attitudes on the topic.

In 2017, some insurers’ policies were revealed to include “taking into account an insured’s heredity” [17]. This was explained as an outdated description based on the practices in the 1970s wherein the family histories of applicants were collected. The firms announced that they would remove this from their policies [17]. The first training workshop on genetics and ethics for life insurance employees was held by the Life Insurance Association of Japan in 2018. We asked the workshop participants to complete an anonymous online questionnaire with ten questions to understand their attitudes toward risk selection and regulations. We also recruited interviewees from the survey respondents to collect their personal perspectives. The study was conducted in accordance with the Code of Ethics published by the Japan Sociological Society. Completion of the entire questionnaire was considered as participant’s consent. We obtained informed consent from all the interviewees. As shown in Table 1, 100 of the 102 participants from 41 insurers responded to the questionnaire. Most were in management positions and 87% were male. Thirty respondents agreed to a semi-structured interview; however, nine were selected (anonymized as A to I, respectively), based on their company type and role, to obtain diverse opinions.

**Table 1:** Respondent and interviewee characteristics

	*N*				
Respondents	100				
	%				
Gender					
Male	88				
Female	12				
Age					
30s	23				
40s	53				
50s	17				
Position					
Manager	58				
Senior manager	18				
Excecutive	2				
Section					
Planning	46				
Education	25				
Actuary or product	9				
Underwriting	3				

	*N*				
Interviewee	9				
	Company	Age	Gender	Section	Position
A	Other	50s	Male	Underwriting	Manager
B	Traditional	40s	Male	Planning	Manager
C	Traditional	30s	Male	Planning	Manager
D	Foreign	40s	Male	Education	Senior manager
E	Foreign	50s	Male	Underwriting	Senior manager
F	Other	50s	Female	Product	Manager
G	Other	30s	Male	Planning	Unknown
H	Traditional	40s	Male	Planning	Manager
I	Other	50s	Male	Planning	Senior manager

We questioned the participants to give priority to their concerns on adverse selection. The following concerns were listed in the question, “applicants with the intention of concealing the future illness discovered by a genetic test,” “applicants with the intention of suicide,” “applicants with the intention of murder for insurance claims,” “applicants with the intention of concealing their current physical condition,” “applicants with intention of concealing their current job,” “applicants with the intention of concealing relatives with genetic disorders,” “NA (none of the above),” and the participants were required to rank them as the highest concern, the second highest concern, and the third highest concern. As shown in Fig. 1, the biggest concern (60%) was “applicants with the intention of concealing their current physical condition.” However, “applicants with the intention of concealing the future illness discovered by a genetic test” was also consistently selected as a concern, as was “applicants with the intention of suicide.” Seven of the nine interviewees did not believe that adverse selection has been a significant issue because it does not currently affect insurers’ profitability, despite applicants apparently concealing their genetic results. Mr E stated that large-scale insurers have no problems with adverse selection, as their risks are distributed over a large number of customers and a range of products, such as death benefits and endowments. However, six interviewees reported a high risk of adverse selection for large insurance policies and believed genetic testing should be allowed for future large policies, as is the case in the UK [18].

**Fig. 1:**
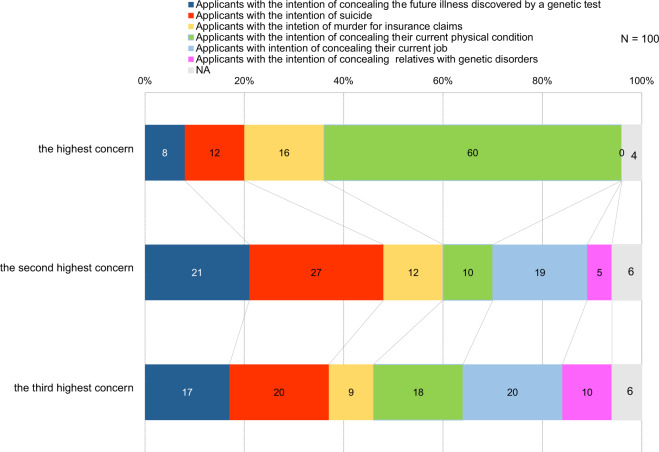
Concerns on adverse selection. Rank selection was used to measure the respondents’ attitudes toward concerns on adverse selection

Some interviewees were concerned about maintaining actuarial fairness if people with genetic risks were automatically insured. Mr D commented that providing the same coverage to those with higher risks would be unfair as to those with lower risks. Mr I opposed insuring people predisposed to future illnesses predicted by genetic testing while rejecting others based on symptoms of diseases. Only one person (Mr G) commented that insurers should accept people with possible future illnesses revealed by genetic tests to protect human rights.

As shown in Fig. 2, regarding regulations preventing insurers from using genetic information, 65% of the respondents wanted insurers to create self-regulatory guidelines. In addition, five interviewees opposed laws against using genetic information. Mr A argued that it was unreasonable to legally restrict insurers’ freedom of risk selection. Mr B wanted the industry to avoid public discussions and to make adjustments as situations change, such as by creating guidelines and providing in-house education within the industry.

**Fig. 2:**
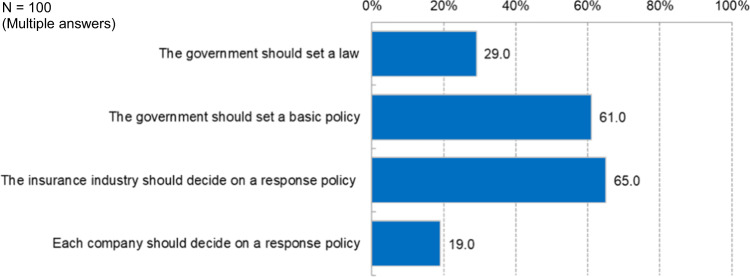
Perceptions on regulations. Multiple answer selection was used to measure the respondents’ attitudes toward perception on regulations

This study has some limitations: we cannot generalize the results due to selection bias. Further, we cannot explain why Japanese insurers have not addressed these issues in the past, as our study included only current employees. However, we provide a general overview of Japanese insurers’ attitudes about requesting genetic information from applicants.

We found that genetic discrimination is not generally considered as a topic of human rights. We also found that insurers have uncertain fears and concerns about adverse selection in terms of actuarial fairness but not regarding profits. In practice, premium differences based on gender, age, and amount are justified in Japan according to actuarial fairness, while this is prohibited in the EU [19, 20]. It is not surprising that the insurers’ perspectives on discrimination have not extended to the genetic status of the insured.

A majority of participants were reluctant to engage the public in open discussions of the issue and would prefer to create internal guidelines for using genetic data. However, we believe that public dialog and consultation are necessary to gain understanding of the people during this process.

## References

[1] Stewart AC (1991). Ethical and social implications of the Human Genome Project: the issue in the UK. Sci Policy.

[2] McGleenan T (2000). Legal and policy issues in genetics and insurance. Community Genet.

[3] Macdonald AS (2003). Moratoria on the use of genetic tests and family history for mortgage-related life insurance. Br Actuarial J.

[4] Viswanathan KS (2007). Adverse selection in term life insurance purchasing due to the BRCA 1/2 genetic test and elastic demand. J Risk Insurance.

[5] Deguchi H. The introduction to life insurance. Tokyo: Iwanami Shoten Publishers; 2009.

[6] Harper PS (1993). Insurance and genetic testing. Lancet.

[7] Pokorski RJ (1995). Genetic information and life insurance; key issues regarding use of genetic information (second of two parts). J Insurance Med.

[8] Ruth C, Ngwena C (1995). The human genome project, predictive testing and insurance contracts: ethical and legal responses. Res Publica.

[9] Raeburn S (2002). Implications of genetic testing for the insurance industry: the UK example. Community Genet.

[10] HM Government and the Association of British insurance. Code on genetic testing and insurance. 2018. https://assets.publishing.service.gov.uk/government/uploads/system/uploads/attachment_data/file/751230/code-on-genetic-testing-and-insurance.pdf. Accessed 28th Aug 2020.

[11] Tiller J, Morris S, Rice T, Barter K, Riaz M, Keogh L (2019). Genetic discrimination by Australian insurance companies: a survey of consumer experiences. Eur J Hum Genet.

[12] The Financial Services Council. FSC announces moratorium on genetic tests for life insurance to start in July 2019. 2018. https://www.hgsa.org.au/hgsanews/fsc-announces-moratorium-on-genetic-tests-for-life-insurance. Accessed 28th Aug 2020.

[13] Japan Institute of Life Insurance. Survey on life insurance in 2018. Tokyo: Japan Institute of Life Insurance; 2018.

[14] McEwen JE, McCarty K, Reilly PR (1993). A survey of medical directors of life insurance companies concerning use of genetic information. Am J Hum Genet.

[15] The Mainichi Newspaper. Osaka morning edition. Considering the use of genetic diagnosis for life insurance underwriting, more than half of the 26 major insurers in Japan. 2000.

[16] Harada K. Research on the collection and use of genetic information in life insurance applications. Master’s thesis. Ochanomizu University. 2018.

[17] The Asahi Shimbun. Tokyo morning edition. Genetic information on 33 Japanese insurer’s insurance policy. 2017.

[18] HM Government and Association of British Insurance. Concordat and moratorium on genetic and insurance. 2005. https://www.abi.org.uk/globalassets/sitecore/files/documents/publications/public/2014/genetics/concordat-and-moratorium-on-genetics-and-insurance.pdf. Accessed 28th Aug 2020.

[19] EU Charter of Fundamental Rights Article 23. Equality between women and men must be ensured in all areas, including employment, work and pay. https://fra.europa.eu/en/eu-charter/article/23-equality-between-women-and-men. Accessed 28th Aug 2020.

[20] The Council of the Europe Union. Implementing the principle of equal treatment between men and women in the access to and supply of goods and services. 2004.

